# Surveillance of *Ixodes ricinus* ticks (Acari: Ixodidae) in Iceland

**DOI:** 10.1186/s13071-017-2375-2

**Published:** 2017-10-10

**Authors:** Matthias Alfredsson, Erling Olafsson, Matthias Eydal, Ester Rut Unnsteinsdottir, Kayleigh Hansford, William Wint, Neil Alexander, Jolyon M. Medlock

**Affiliations:** 1 The Icelandic Institute of Natural History, Urridaholtsstraeti 6–8, 212 Gardabaer, Iceland; 2 Institute for Experimental Pathology at Keldur, University of Iceland, Keldnavegur 3, 112 Reykjavik, Iceland; 3 Emergency Response Department, Medical Entomology Group, Public Health England, Porton Down, Salisbury, Wiltshire UK; 4 Department of Zoology, Environmental Research Group Oxford, University of Oxford, Oxford, UK; 5 NIHR Health Protection Research Unit in Emerging and Zoonotic Infections, PHE Porton Down, Porton Down, Salisbury, UK

**Keywords:** Surveillance, Tick, *Ixodes ricinus*, Iceland

## Abstract

**Background:**

*Ixodes ricinus* is a three-host tick, a principal vector of *Borrelia burgdorferi* (*s.l.*) and one of the main vectors of tick-borne encephalitis (TBE) virus. Iceland is located in the North Atlantic Ocean with subpolar oceanic climate. During the past 3–4 decades, average temperature has increased, supporting more favourable conditions for ticks. Reports of *I. ricinus* have increased in recent years. If these ticks were able to establish in a changing climate, Iceland may face new threats posed by tick-borne diseases.

**Methods:**

Active field surveillance by tick flagging was conducted at 111 sites around Iceland from August 2015 to September 2016. Longworth mammal traps were used to trap *Apodemus sylvaticus* in southwestern and southern Iceland. Surveillance on tick importation by migratory birds was conducted in southeastern Iceland, using bird nets and a Heligoland trap. *Vulpes lagopus* carcasses from all regions of the country were inspected for ticks. In addition, existing and new passive surveillance data from two institutes have been merged and are presented. Continental probability of presence models were produced. Boosted Regression Trees spatial modelling methods and its predictions were assessed against reported presence.

**Results:**

By field sampling 26 questing *I. ricinus* ticks (7 males, 3 females and 16 nymphs) were collected from vegetation from three locations in southern and southeastern Iceland. Four ticks were found on migratory birds at their arrival in May 2016. A total of 52 *A. sylvaticus* were live-trapped but no ticks were found nor on 315 *V. lagopus* carcasses. Passive surveillance data collected since 1976, reports further 214 *I. ricinus* ticks from 202 records, with an increase of submissions in recent years. The continental probability of presence model correctly predicts approximately 75% of the recorded presences, but fails to predict a fairly specific category of recorded presence in areas where the records are probably opportunistic and not likely to lead to establishment.

**Conclusions:**

To the best of our knowledge, this study represents the first finding of questing *I. ricinus* ticks in Iceland. The species could possibly be established locally in Iceland in low abundance, although no questing larvae have yet been detected to confirm established populations. Submitted tick records have increased recently, which may reflect an increase in exposure, or in interest in ticks. Furthermore, the amount of records on dogs, cats and humans indicate that ticks were acquired locally, presenting a local biting risk. Tick findings on migratory birds highlight a possible route of importation. Obtaining questing larvae is now a priority to confirm that *I. ricinus* populations are established in Iceland. Further surveys on wild mammals (e.g. *Rangifer tarandus*), livestock and migratory birds are recommended to better understand their role as potential hosts for *I. ricinus*.

**Electronic supplementary material:**

The online version of this article (10.1186/s13071-017-2375-2) contains supplementary material, which is available to authorized users.

## Background

Ticks are important vectors of arthropod-borne pathogens in Europe and a major threat to human and animal health [1]. Over recent years, ticks have increased in abundance and expanded their distribution limits within Europe [2] both in their altitudinal and latitudinal range [3]. Distribution of ticks to regions that were previously considered free of ticks and their associated pathogens are now facing new threats posed by tick-borne diseases [4–6].

*Ixodes ricinus* (Acari: Ixodidae) is a three-host tick with three active life stages, larva, nymph and adult [7]. The immature stages are found on hosts of all sizes while adult stages tend to be the only stage found on larger hosts [3]. This tick species can transmit a large variety of pathogens of medical and veterinary importance [3]; it is the principal vector of *Borrelia burgdorferi* (*s.l.*) (agent of Lyme borreliosis) and one of the main vectors of Tick-borne encephalitis (TBE) virus. It is also a known and potential vector for other pathogens such as *Babesia*, *Anaplasma*, *Rickettsia*, *Borrelia miyamotoi* and louping ill virus [8–10].

*Ixodes ricinus* is an ectothermic but strongly temperature dependent tick [11] and its activity and survival depends on the degree of relative humidity and saturation deficit. Therefore, changes in global climate might affect the geographic distribution of this species [12, 13]. Other environmental variables such as the assemblage and abundance of hosts and landscape characteristics are also important [14, 15] in sustaining populations and in providing suitable microclimate. Northern tick species such as *I. ricinus*, are adapted to sub-zero temperatures (via diapause), but enhanced snow cover might promote overwintering tick survival as it prevents repeated freeze and thaw and can keep ground temperatures above zero [3]. Swedish studies [16, 17] have shown a close correspondence between the distribution of *I. ricinus* and the duration of the vegetation and snow cover periods. If snow cover period is more than 150 days per year and mean daily temperature is lower than 5 °C for more than 170 days, it is unlikely for ticks to establish in that area. Survival rate of small mammals over cold winters is important for ticks [18]. Winters in Iceland are becoming milder and spring and autumn warmer with higher humidity. These changes could further enable ticks to establish and increase in abundance in new localities [19–21].

Ticks are commonly found on migratory birds and their continental dispersal by birds is well known [22–24]. This seems to be the most likely route for ticks to extend their distribution to remote islands in northern Europe.

This paper considers further evidence for the latitudinal spread of *I. ricinus* in northern Europe. Other recent work has focussed on northward expansion of the species in Norway [5, 6], Sweden [4] and Faroe Islands [25].

Iceland is located in the North Atlantic Ocean, between Greenland, the British Isles and Norway. It is a geologically young island on the divergent boundary on the Mid-Atlantic Ridge. The land area is approximately 103,000 km^2^ with population of 332,500 people (January 2016, Statistics Iceland). Iceland was settled by humans during the late tenth century. The first settlers brought livestock, mostly sheep and horses, and there is a tradition of free ranging sheep farming during the summer. The climate is subpolar oceanic but varying between different parts of the island. The warm North Atlantic current provides overall warmer annual temperature than in other areas of similar latitude [26].

Icelandic fauna is rather poor, with the exception of migrating birds. The arctic fox (*Vulpes lagopus*) is the only native wild mammal found in Iceland. Other mammals such as reindeer (*Rangifer tarandus*), mink (*Mustela vison*), European rabbit (*Oryctolagus cuniculus*), wood mouse (*Apodemus sylvaticus*), house mouse (*Mus musculus*), brown rat (*Rattus norvegicus*) and common livestock have all been introduced (the mice by the first settlers over 1100 years ago) [27]. Domestic cats and dogs are common in towns, villages and farms in the countryside. Around 75 bird species commonly breed in Iceland, 12 of which are passerine species. Of those twelve, the following species migrate: wheatear (*Oenanthe oenanthe*), white wagtail (*Motacilla alba*), meadow pipit (*Anthus pratensis*) and redwing (*Turdus iliacus*) [28].

Eight tick species of the family Ixodidae have been identified and published in Iceland: *Ixodes uriae*, *I. caledonicus*, *I. ricinus*, *I. hexagonus*, *I.* cf. *scapularis*, *Rhipicephalus sanguineus*, *Dermacentor variabilis* and *Hyalomma aegyptium* [29]. Only *I. uriae* and *I. caledonicus* are considered to be established in Iceland [29].

The first report of *I. ricinus* in Iceland is from 1967 when a nymph was found on an *Anthus pratensis* (meadow pipit) (wrongly named *Anthus trivialis* in the publication) in Surtsey [30] but the specimen was taken to Sweden for identification and is presumably preserved there. Reported cases on passive tick surveillance have been collected by the Icelandic Institute of Natural History (IINH) and the Institute for Experimental Pathology at Keldur, University of Iceland (IEPKUI) since 1976. This paper details the results of an improved surveillance scheme, merging the two former schemes together, and reports the first field-based survey of likely habitats of *I. ricinus* in Iceland. This survey is a part of an active surveillance programme funded by the European Food Safety Authority (EFSA) and the European Centre for Disease Prevention and Control (ECDC) VectorNet project.

## Methods

To detect the presence of questing *I. ricinus*, various strategies were employed. A nationwide field survey of likely tick habitats was conducted and active surveillance was established. Furthermore, mass media was used to encourage veterinarians, health-care workers and the public to collect ticks and submit them to the passive surveillance scheme.

Both IINH and IEPKUI have separate collections of ticks that have been sent to the institutes. These collections contain useful information such as dates of findings, locations and hosts. These data were used to identify areas of likely suitability for surveillance of questing ticks.

Active field surveillance was conducted from August 2015 to September 2016. Priority was given to southern and southwestern Iceland on account of climate suitability, proximity to the main urban centres around Reykjavik, the greater concentration of passive reports, migratory bird arrival landfall sites and the proximity to IINH/IEPKUI and travel networks.

In total, 111 localities were surveyed. Of those, 25 localities were visited more than once. During late August (24th–28th) 2015, 37 localities in southern and southwestern Iceland were surveyed to coincide with the highest number of tick reports. In September (25th–27th) 2015 a further 17 sites were surveyed in eastern Iceland and inland around Hallormsstadarskogur, in areas where ticks have been reported. In 2016 field surveillance was continued and 86 locations were surveyed around Iceland from May (7th) to late August (28th). These localities constitute all of the main woodland sites. Additionally, camping sites, picnic areas, community parks and coastal grasslands were also surveyed.

**Fig. 1:**
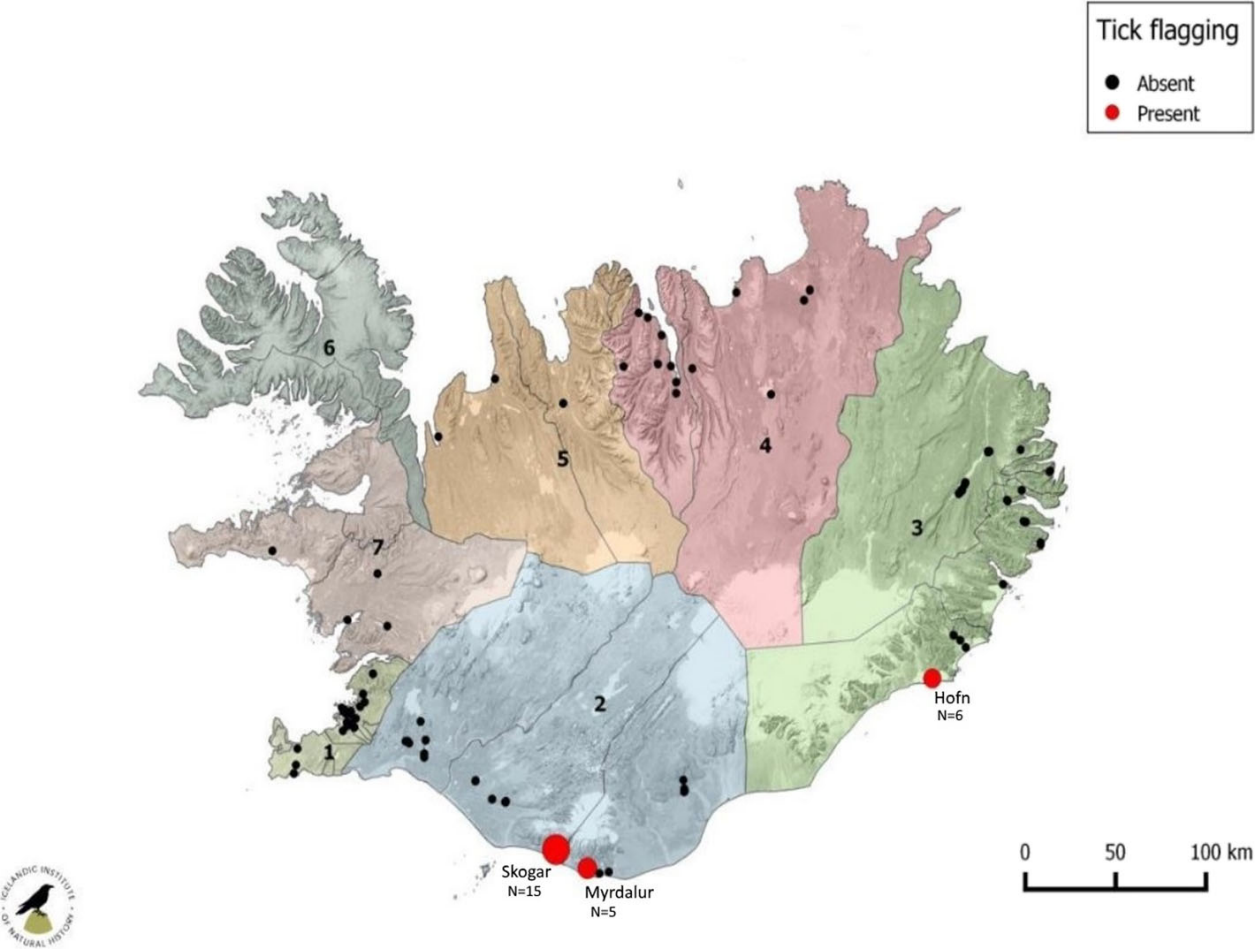
Locations of sites in Iceland that were surveyed for *Ixodes ricinus* in 2015 and 2016. The seven research areas shown by different colours (see text)

Tick flagging [31] was conducted at each location. A 1 m^2^ cotton cloth was dragged over a distance of 5 m at a slow walking pace. The habitat was sampled at random with 45 separate 5-m drags conducted at each location. Ticks collected were stored in tubes filled with 80% ethanol and were taken back to the laboratory for identification using the keys of Hillyard [31]. A full list of sites visited is detailed in Additional file 1: Table S1, their locations are shown in Fig. 1.

Small mammal surveys were conducted in August (24th–28th) 2015 with 55 Longworth live-traps set for wood mice over four consecutive nights in a woodland edge area in Heidmork. In October (25th–28th) 2016 small-mammal surveys were continued in a mixed woodland area in Skogar, where ticks have been reported on cats, dogs and humans. Additionally, 315 carcasses of arctic foxes legally hunted in all regions of the country were inspected from August 2015 to September 2016.

Surveillance on migratory birds was conducted for 3 days from 6th–8th May 2016 at Hofn, southeastern Iceland to investigate which species are carrying ticks to Iceland and the abundance of ticks on birds. Bird nets and a Heligoland trap were used to trap the birds.

The continental probability of presence models were produced using the VECTORNET Gap Analysis procedures set out in [32] and summarised as follows: (i) 13,000 locations with known presence were identified from the VECTORNET archive consisting of both administrative unit and point location records; (ii) an equal number of absence locations were defined using a habitat suitability mask based on land cover types and environmental limits based on literature and expert opinion (unsuitable = absent). Boosted Regression Trees spatial modelling methods were used to establish a statistical relationship between tick presence and a wide range of demographic, climatic and remotely sensed covariates, which was then used to produce the predicted presence map. The remotely sensed covariates were based on Temporal Fourier Processing of a 15 year time series of MODIS satellite imagery providing biologically relevant descriptors relating to day and night-time land surface temperature and vegetation indices [33]. The modelling was implemented through the VECMAP software suite.

## Results

### Passive surveillance

**Fig. 2:**
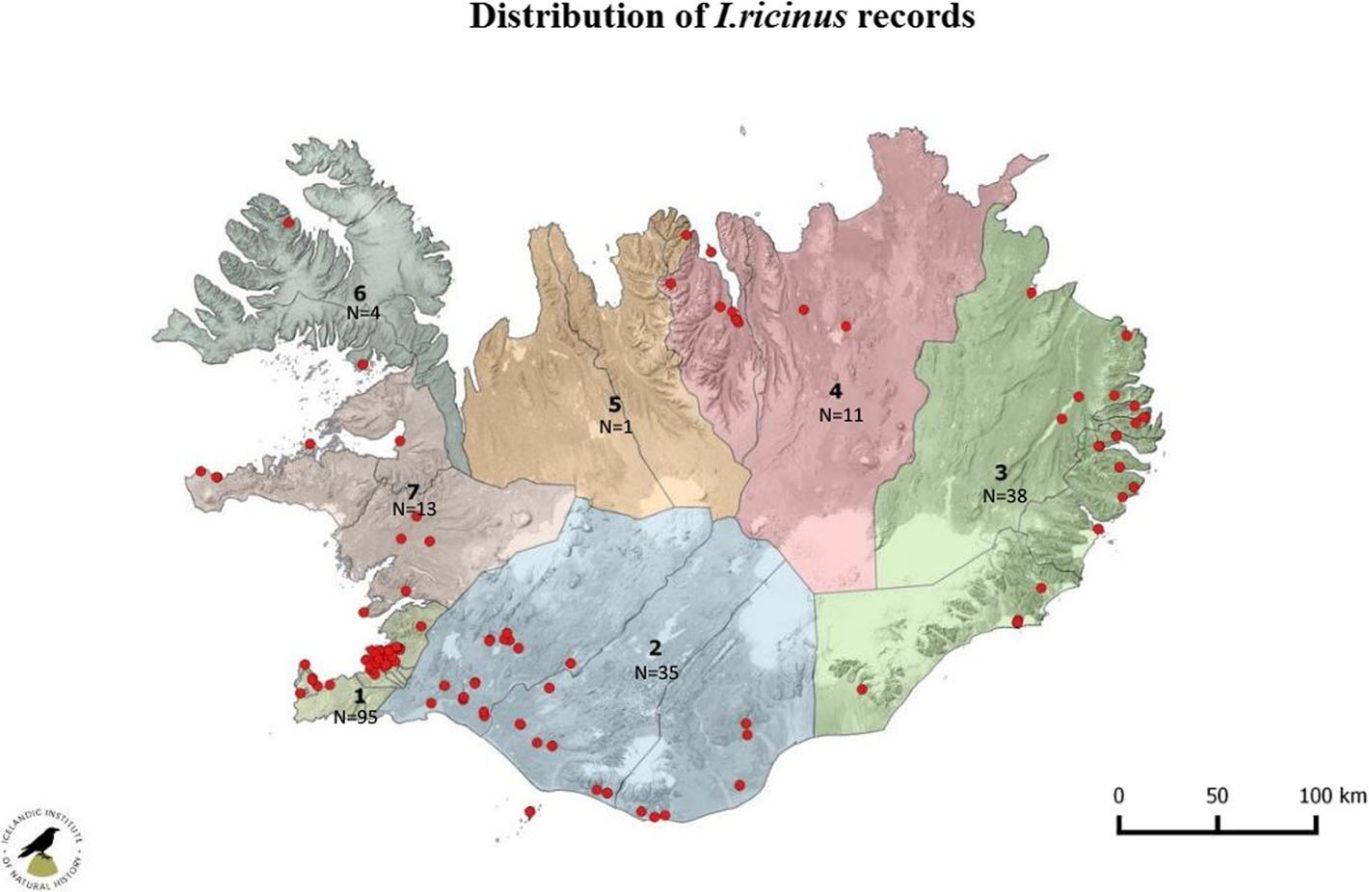
Distribution of *I. ricinus* in Iceland through passive surveillance. Total number of tick records are shown for each area (*N*)

**Fig. 3:**
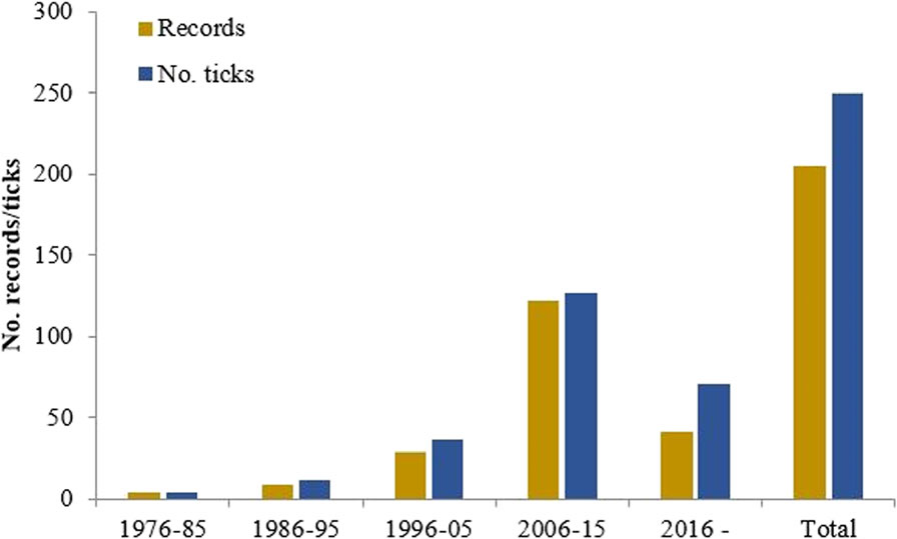
Total number of *I. ricinus* records and ticks in Iceland between 1976 and 2016

In total 202 records and 214 *I. ricinus* ticks have been found and identified in Iceland through the passive surveillance scheme during 1976–2016 (including historical reports from 2012 to 2016, which have not been reported previously). These records were distributed all over Iceland but most of them came from southwestern and eastern Iceland (Fig. 2). Of these 214 ticks, 177 were female, two were male, 25 nymphs and 7 larvae. Three specimens of *I. ricinus* could not be identified as females or nymphs as they were in poor condition. The submission of tick records has increased over the years (Fig. 3) and seasonal occurrence of these records indicate a peak in August (Table 1). In these records dogs were the most common hosts for *I. ricinus* followed by cats and humans.

**Table 1:** Seasonal occurrence (when known) of *I. ricinus* in Iceland recorded through passive surveillance from 1976 to 2016

Month	Females	Males	Nymphs	Larvae	Total
May	6		5	7	18
June	7		1		8
July	26		7		33
August	64	1	4		69
September	42		4		46
October	16				16
November	3	1			4
Total	164	2	21	7	194

Most tick records came from Area 1 (consisting of the capital area, Kjosarsysla and Gullbringusysla) in southwestern Iceland. Ninety-five records were collected in the years 1976–2016 including 88 females, 11 nymphs and seven larvae. Dogs were the most common hosts for *I. ricinus* with 57 records, followed by cats (*n* = 14) and humans (*n* = 12). One record represented five nymphs and seven larvae collected from a wheatear (*Oenanthe oenanthe*). A further eight records were associated with humans and dogs that had travelled abroad. Eleven records had no data on hosts.

In Area 2 (Arnessysla, Rangarvallasysla, Vestur-Skaftafellssysla and Vestmannaeyjar counties) in southern Iceland, 35 records (1980–2016) of *I. ricinus* have been collected. Of these, 25 were females, one male and six were nymphs, but three samples were damaged so they could not be identified. Dogs were the most common hosts (*n* = 20) followed by humans (*n* = 8), cats (*n* = 4) and sheep (*n* = 2). One record had no host data. Two records (female and a nymph) were linked to human travel.

A total of 38 records (1977–2016) came from Area 3 (Austur-Skaftafellssysla, Sudur-Mulasysla and Nordur-Mulasysla) in eastern Iceland, consisting of 30 female, one male and seven nymphs. Cats were the most common hosts (*n* = 13) then dogs (*n* = 11), humans (*n* = 11), reindeer (*n* = 1) and sheep (*n* = 1). One record had either cat or dog as a host. Two females records were associated with human travel.

Eleven records (1991–2016) were from Area 4 (Nordur-Thingeyjarsysla, Sudur-Thingeyjarsysla and Eyjafjardarsysla) in northeast Iceland, consisting of 11 females and one nymph. Dogs (*n* = 5), cats (*n* = 3), humans (*n* = 2) and sheep (*n* = 1) were the reported hosts. Two records of female ticks were found on dogs in quarantine.

There is only one record (2005) from Area 5 (Skagafjardarsysla, Vestur-Hunavatnssysla and Austur-Hunavatnssysla) in northern Iceland; a female tick attached to a dog.

Four records (2006–2015) were reported from Area 6 (Strandasysla, Nordur-Isafjardarsysla, Vestur-Isafjardarsysla, Vestur-Bardastrandarsysla and Austur-Bardastrandarsysla) in northwestern Iceland. All records were females; three on dogs, and one on a cat.

In total, 13 records (2000–2016) were reported from Area 7 (Dalasysla, Snaefellsnessysla, Myrasysla and Borgarfjardarsysla) in western Iceland. All records were female ticks; on dogs (*n* = 8), cats (*n* = 4) and a human (*n* = 1). A further five records (1986–2002) of *I. ricinus* had no information on location.

### Active surveillance

In August 2015, 37 field sites were surveyed in southern and southwestern parts of Iceland. In September 2015, a further 17 sites were surveyed in the East. In 2016, 86 suitable tick habitats around Iceland were surveyed, including some of the sites visited in 2015.

**Fig. 4:**
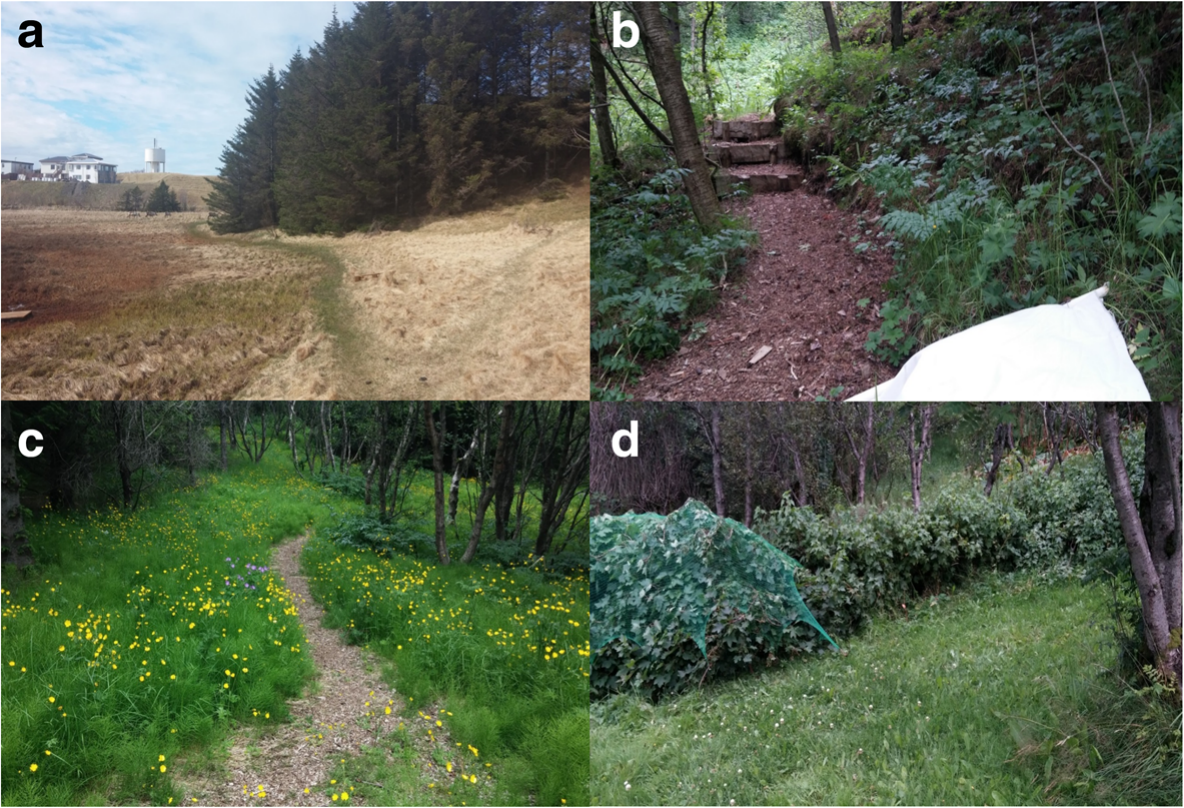
Questing *I. ricinus* ticks were found at three separate locations in Iceland. **a** Hrossabithagi at Hofn. **b, c** Skogar. **d** Farm in Myrdalur

In 2015, no questing ticks were found. However on 7th May 2016 six questing nymphs were found in Hrossabithagi at Hofn in southeastern Iceland (Fig. 4a). Reindeer were present at this location and ticks were found in close proximity. These were the first questing ticks that have been found in Iceland and they were identified as *I. ricinus*.

On the 24th June 2016 four questing *I. ricinus* were found at Skogar, southern Iceland (Fig. 4b, c) (one male and three nymphs). On 19th August, Skogar was revisited and 11 individuals of *I. ricinus* were found (three males, two females and six nymphs). On the same day, a farm in Myrdalur was surveyed because of a recent tick record. Five *I. ricinus* were found (three males, one female and one nymph); all found next to redcurrant (*Ribes rubrum*) bushes (Fig. 4d).

Surveillance of ticks on migratory birds was conducted over 3 days in May (6th–8th) at Hofn. One *I. ricinus* nymph was found on a redwing (*Turdus iliacus*) and three *Ixodes* larvae on a meadow pipit (*Anthus pratensis*) but the larvae could not be identified to species level. At that time most of the migratory birds had already arrived and only 10 birds were captured.

In August 2015 small mammal surveys were conducted in Heidmork woodland in southwest Iceland. No mammals were collected during the August trapping. In October (25th–28th) 2016 small mammal surveys, based on the amount of questing ticks, were conducted at Skogar, in the hope of finding tick larvae. Over three nights, a total of 52 wood mice were caught and inspected but no ticks were found. Carcasses of arctic foxes are being sent to IINH all year round for inspection. No ticks were found on 315 foxes inspected, hunted from August 2015 to September 2016. Seasonal occurence of ticks found through active surveillance is shown in Table 2.

**Table 2:** Seasonal occurrence of *I. ricinus* in Iceland from active surveillance from 2015 to 2016

Month	Females	Males	Nymphs	Larvae	Total
May			7	3	10
June		1	3		4
July					0
August	3	6	7		16
September					0
October					0
November					0
Total	3	7	17	3	30

### Validation of spatial distribution model

**Fig. 5:**
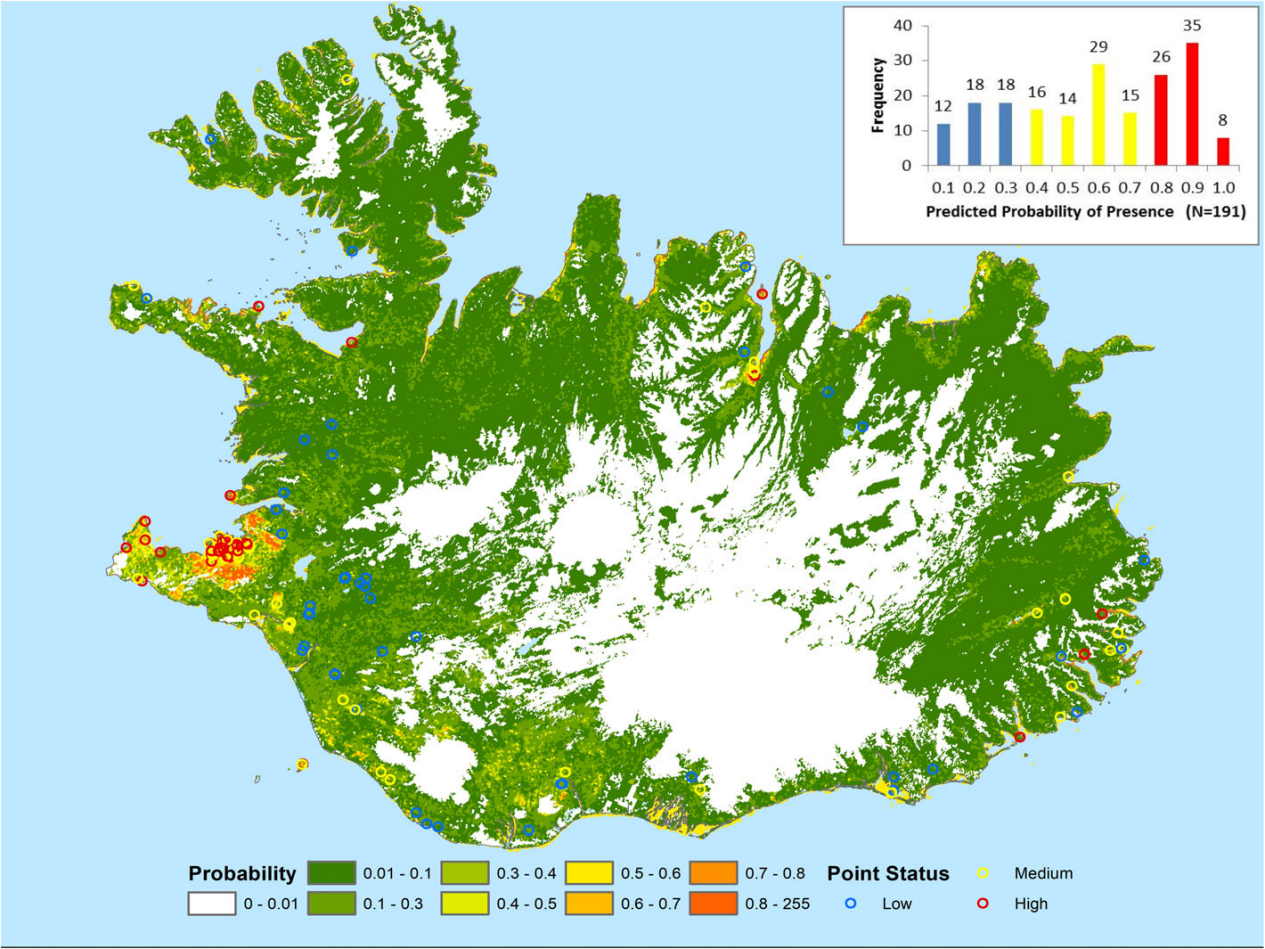
Distribtion model for Iceland with the presence records presented as circles. Circle colour relates to predicted probability of presence (< 0.3, Absent; 0.3–0.5, Possible; > 0.5, Present). Inset: Histogram of predicted propabilities for the recorded presence points

The occurrence data collected by IINH/IEPKUI provided the opportunity to assess the extent to which spatial models produced for continental Europe by the ECDC and EFSA funded VECTORNET project could be used for Iceland, which is located at the northern extreme of the model extent. The model for Iceland is shown in Fig. 5, with the presence records presented above overlaid as circles. Circle colour relates to predicted probability of presence (< 0.3, Absent; 0.3–0.5, Possible; > 0.5, Present). Figure 5 also shows the histogram of predicted probabilities for the recorded presence points.

These results show that three quarters have predicted values of more than the 0.3 threshold identified from a ROC curve based on the recorded presences and an equal number of the closest absences taken from the probability model (AUC 0.886, 95% CI: 0.854–0.916, *n =* 382). The lowest values (< 0.3) which represent the least accurate predictions are shown as blue circles in Fig. 5 can be seen to be largely restricted to the south western hinterland. If the medium and high categories of presence are taken to be present, these patterns suggest the model provides good indication of the areas that show the highest density of recorded presence points and only fails to predict a fairly specific (and clustered) category of recorded presence.

## Discussion

Established populations of *Ixodes ricinus* in Iceland has not yet been confirmed. For establishment, *I. ricinus* requires favourable habitats with a required density of suitable hosts for all tick stages and a climate favourable to survive the winter. Historical records indicate that *I. ricinus* is acquired locally in Iceland and the number of records through the passive schemes have increased over recent years. The data reported in this study include important information such as locations, dates, hosts and travel history. Many records of engorged female *I. ricinus* ticks on dogs and cats show that these animals are being exposed to ticks through the environment (running free in wild vegetation). The majority of all records were acquired in Iceland since only a few tick records have been associated with foreign travel, both on humans and companion animals. The lack of ticks associated with travel may be explained by the strict protocols for treating animals entering Iceland (at present 4 weeks quarantine), which reduces the risk of tick importation to the environment.

The historical data show that *I. ricinus* records were few from 1976 to 2004 but have increased since then [29]. The data also indicate a peak of records, from August to October and most of them were gathered in southwestern and eastern Iceland. With this information in mind it was decided to focus on these two areas in 2015. There are a few large woodlands in Iceland that could provide a suitable habitat for *I. ricinus*. However, the likelihood of ticks finding hosts in these woodlands are limited. In rural woodlands, wood mouse, sheep, European rabbit and birds are potential hosts. In heathers and open areas, arctic fox, wood mouse and reindeer as well as free ranging sheep could act as a potential hosts. In urban areas, dog, domestic cat, wood mouse, horse, mink and brown rat would be possible hosts, even the European rabbit, where present. All these mammals may, however, exist in too low a density in order to sustain viable tick populations. So far only one tick has been found on a wild mammal (reindeer). Further animal surveys are recommended. Only four records of *I. ricinus* have been found on sheep and there is no further evidence to suggest that livestock are important tick hosts in Iceland, but further surveys are recommended.

There are no native conifer woodlands in Iceland but plantations of evergreen species mixed with deciduous trees could provide ticks the leaf litter and moist environment needed, in order to survive the winter. The variety of herbs that are commonly found in woodland tick habitats elsewhere in northwest Europe is not similar in the Icelandic woodlands surveyed. Instead the ground and herb vegetation in Iceland is commonly characterised by dominant dense grass that conserves humidity well in leaf litter. Tick findings on migratory birds might indicate a likely route of importation in the spring each year. Migratory passerine birds such as wheatear, white wagtail, meadow pipit, and redwing are the most likely bird species to transport ticks to Iceland. These birds travel to Iceland from northern Africa, western Europe and the British Isles [28]. When arriving in Iceland the majority of birds first stop in southern, southeastern and eastern parts of Iceland before they disperse to other parts of the country [28]. Inspecting migratory birds for ticks is important in understanding the role that migratory birds may play in introducing ticks into Iceland. Collaboration with Fuglaathugunarstod Sudausturlands (a bird observatory) at Hofn will provide valuable information about tick infestation on birds and which bird species carry ticks to Iceland. Finding four ticks on migratory birds at the beginning of May, when most of the birds had already arrived, emphasises the importance of further survey to properly assess tick infestation rates.

Ticks brought to Iceland may be able to moult during the spring and summer and then find an animal or human in the autumn, or the following spring if they survive the winter. Dogs and cats may play a role in feeding local tick populations in Iceland. These pets are more abundant around the main towns and cities and therefore it seems most likely for ticks to find pets close to Reykjavik in southwestern Iceland. However, congregations of migratory birds are mostly in southern and southeastern Iceland where they encounter many more areas of woodland where there are few pets and wild mammal densities are low. Wood mice are abundant in woodlands though and therefore small mammal surveys are important. Livestock and reindeer roam widely in rural areas of Iceland. The reindeer populations is present locally in southeastern and eastern Iceland only. They stay in open areas in the highlands over the summer and autumn but during winter and spring they are frequently seen in villages such as Hofn and Eskifjordur. Some smaller herds of immature individuals tend to stay in lowland sites all year round [34]. There is only one record of *I. ricinus* on reindeer but the role of reindeers as hosts for ticks has never been investigated. Sheep are mostly located in open areas but also graze in birch groves with luxuriant undergrowth. There are four records of *I. ricinus* found on sheep. So they could possibly serve as hosts for ticks. *Ixodes ricinus* could be transferred with migrant birds such as wheatears, meadow pipits or redwings into the outfield where livestock is present. Nevertheless, although that may support tick populations in the outfield with the help of wood mice, birds and livestock, establishment seems unlikely. In fact there are few woodlands in southern and southeastern Iceland that could provide a suitable habitat for *I. ricinus* with congregations of migratory birds where ticks can find hosts. Skogar is a mixed woodland where conifers have been planted within a native birch wood next to Skogafoss; a popular tourist attraction. A walking path where people walk their dogs goes through the woodland. At Hofn in southeastern Iceland there are few isolated conifer plantations where congregations of migratory birds occur similar to that reported in the Faroe Islands [25]. Hrossabithagi is one of those woodlands, surrounded by either grassland or wet meadow that is being grazed and reindeer are known to stay in the area in the spring.

Climate could possibly be a limiting factor for *I. ricinus* in some parts of the country but summer months are certainly warm enough for *I. ricinus* to survive. Temperature during other seasons, winter, spring and autumn, is more likely to be limiting. Data from the Icelandic Meterological Office (1997–2015) show that in the southern parts of Iceland (Reykjavik and Hofn), mean temperature over the winter was rarely below zero while in the northern part (Akureyri) the mean temperature over the winter was usually below zero. Mean temperature over the autumn and spring was always well above zero in Reykjavik, Hofn and Akureyri. Therefore, it is more likely for ticks to survive the winter and for tick populations to establish locally in the southern parts of Iceland rather than in the north. Snow coverage can provide *I. ricinus* humidity and protection from freezing when temperature drops below zero degrees, especially where leaf litter is present. Also according to data from the Icelandic Meteorological Office, snow cover never reached 150 days/year in the years 2000–2015. Local climate can also affect overwintering survival of mammals (potential hosts for *I. ricinus*). For example, estimated survival rate for wood mice during the winter in Iceland is quite low, around 60% per month in woodlands, even lower in open areas [35]; this could be a crucial factor for *I. ricinus*.

Despite significant effort in August 2015, when 54 locations were surveyed at the most likely time of year to encounter ticks, no questing *I. ricinus* were found. Small mammal surveys did not result in any tick findings either but this may have been too early for mammal trapping. Thus, it has been decided to repeat the mice suveys in the future. No ticks were found on arctic fox carcasses. It is certainly unlikely to find ticks on carcasses or in the bags they were stored in. Still this will be part of the tick surveillance in Iceland from now on. Detecting larvae is crucial to confirm establishment of *I. ricinus* in Iceland. Finding questing ticks before the arrival of migratory birds would also indicate overwintering survival of the species, but not necessarily confirm establishment. The lack of small mammal species such as *Myodes* and *Microtus* which are known to be crucial in feeding larval *I. ricinus* [25] and the absence of squirrels and game birds other than ptarmigans (*Lagopus mutus*), further limit host availability. It would be interesting and worthwhile to investigate the role of brown rats (in urban areas) and wood mice in feeding immature stages.

The first questing ticks were found in Hrossabithagi at Hofn under Sitka spruce (*Picea sitchensis*). Reindeers were lying in this area moments before it was flagged so we recommend surveys on reindeer during the hunting season (late summer and autumn). Hrossabithagi seems to be a suitable habitat for *I. ricinus*, also being the first stop for various species of migratory birds. The grassland and wet meadow surrounding the woodland is used for grazing, companion animals, reindeer and humans are present most of the season, wood mice as well. After finding questing ticks at Hrossabithagi, local clinics and veterinarians were asked if they had encountered any ticks this spring. Three tick records had been reported, two on humans, one on dog, all specimens were discarded. On 24th June, Skogar was surveyed and four questing *I. ricinus* ticks were found. This is a herb-rich forest and the ground and herb vegetation consists mostly of wild angelica (*Angelica sylvestris*), common lady’s mantle (*Alchemilla vulgaris*), wood cranesbill (*Geranium sylvaticum*), meadow horsetail (*Equisetum pratense*) and meadow buttercup (*Ranunculus acris*). Human traffic through this woodland is high and there are records of ticks on humans, dogs and cats from Skogar. Furthermore, migratory birds are present in the woodland as well as wood mice. Cows and sheep are present in a grassland nearby and can easily access the woodland. Skogar was revisited on 19th August and 11 ticks were found in a small area. The presence of both female and male ticks, would make it easier for the ticks to produce fertilized eggs to establish a tick population. It is our belief that of the locations we checked in Iceland, Skogar is the most likely place that could sustain a tick population.

In 2016, few reports of *I. ricinus* came from Myrdalur, close to Skogar. One of these records was a tick attached to woman’s leg and she was certain that she had ticks in her garden. This was a large garden with a black cottonwood (*Populus trichocarpa*) plantation, high grass vegetation and redcurrant (*Ribes rubrum*). Five ticks were found by flagging, all of which were found in grass under the redcurrant. This was surprising but possibly ticks had dropped off redwings that visited the redcurrant bush, for the berries. Indeed, a few redwings were seen in the nearby area while flagging. No further ticks were found in other areas surveyed around Iceland. This indicates that tick reports, especially in northern and western Iceland could be opportunistic rather than from a defined tick endemic area. As mentioned before, the herb-rich woodland at Skogar provides many factors that could favour *I. ricinus* needs to establish population such as congregation of migratory birds (that can bring in new ticks each year), humidity, access to various hosts and presence of both male and female ticks. It appears from the amount of ticks and the presence of different life stages that *I. ricinus* might be established locally only in southern Iceland, albeit at low abundance although further evidence for questing larvae is lacking. Furthermore, though in general, Icelandic nature and climate seem rather hostile for ticks, there might already be a small population at Skogar in south Iceland. Such a pattern would certainly be consistent with the spatial modelling validation results: with the model able to predict the areas where the tick may be locally established, but not able to identify the opportunistic occurrences in sites where the vector does not become established.

For this reason, Iceland has now established active surveillance on *I. ricinus* that is led by IINH and IEPKUI. Passive surveillance has improved greatly with the collaboration of these two institutes and with assistance from veterinarians, healthcare workers and the public. Tick flagging will be continued in woodlands in southwestern, southern and southeastern parts of Iceland (especially Skogar) and the main focus will be on finding larvae to confirm establishment of *I. ricinus*. Sampling reindeer and livestock would provide important information and is recommended. As part of finding larvae, small-mammal trappings will be conducted at Skogar and nearby areas. This will also improve the understanding of the potential role of wild mammals for the tick populations. Surveillance on migratory birds has been established at Hofn bird observatory station and all migratory birds captured will be checked for ticks from now on. In the near future questing ticks found in Iceland will be tested for pathogens, a necessary procedure to gain understanding on the risk posed by the presence of *I. ricinus* in Iceland for public and animal health.

## Conclusions

Passive surveillance on ticks in Iceland was established in 1976, with numbers of submissions increasing over time. An active surveillance was started in 2016 with 111 sites surveyed and 26 questing *I. ricinus* ticks found at three locations. These were the first findings of questing ticks in Iceland. Preliminary sampling of migratory birds in spring found four ticks on ten birds checked. Mammals were also sampled for ticks with no ticks found on 52 wood mice at Skogar, southern Iceland, nor on 315 inspected Arctic fox carcasses. So far there is no evidence to confirm that *I. ricinus* is established in Iceland; however, the numbers of questing *I. ricinus* (15) at Skogar might be indicative of a local population that needs to be confirmed with further surveillance. This locality in southern Iceland appears the most suitable habitat for a tick population, owing to the density of suitable hosts for all tick stages and climate favourable to survive the winter. It is also possible that the high density of dogs and cats in the Reykjavik area could also facilitate local establishment. Further reseach is required on the possible role of wood mice as hosts for the larvae; the possible role of European rabbit which are continuing to increase in Icelandic woodlands; and the importation of ticks with migratory birds. All ticks collected are now being tested for various pathogens to assess possible disease risk. It is our prediction that in the future, with a warmer climate, an expansion of woodlands and increasing host density, the numbers of questing ticks will increase with a higher likelihood of establishment in certain areas.

## Additional file

Additional file 1: Table S1. Location of field sampling for *Ixodes ricinus* in Iceland (2015–2016). (DOCX 25 kb)

## Abbreviations

ECDC: European Centre for Disease prevention and Control
EFSA: European Food Safety Authority
IEPKUI: Institute for Experimental Pathology at Keldur, University of Iceland
IINH: The Icelandic Institute of Natural History
PHE: Public Health England
