# Supplementary material for: Surveillance of Ixodes ricinus ticks (Acari: Ixodidae) in Iceland
Source: Parasit Vectors. 2017 Oct 10;10:466. doi: 10.1186/s13071-017-2375-2 (PMC5634879; doi:10.1186/s13071-017-2375-2)
Supplement: Additional file 1: Table S1. — Location of field sampling for Ixodes ricinus in Iceland (2015–2016). (DOCX 25 kb) [file 13071_2017_2375_MOESM1_ESM.docx]

**Additional file 1: Table S1.** Locations of field sampling for *Ixodes ricinus* in Iceland (2015 and 2016)

| **No.** | **Location** | **Area** | **Habitat** | **Lat** | **Long** | **Date surveyed** |
| --- | --- | --- | --- | --- | --- | --- |
| 1 | Heidmork | 1 | Conifer Woodland | 64,056494 | -21,79114 | 24^th^ August 2015 |
| 2 | Heidmork | 1 | Conifer Woodland | 64,054908 | -21,793672 | 24^th^ August 2015 |
| 3 | Ellidaardalur | 1 | Mixed woodland | 64,122049 | -21,842043 | 24^th^ August 2015 |
| 4 | Ellidaardalur | 1 | Mixed woodland | 64,120176 | -21,841915 | 24^th^ August 2015 |
| 5 | Ellidaardalur | 1 | Mixed woodland | 64,116055 | -21,841271 | 24^th^ August 2015 |
| 6 | Geirsnef | 1 | Dog park | 64,129147 | -21,837816 | 24^th^ August 2015 |
| 7 | Oskjuhlid | 1 | Conifer Woodland | 64,125776 | -21,924548 | 25^th^ August 2015 |
| 8 | Oskjuhlid | 1 | Conifer Woodland | 64,127949 | -21,925492 | 25^th^ August 2015 |
| 9 | Ulfarsfell | 1 | Conifer Woodland | 64,152844 | -21,736321 | 25^th^ August 2015 |
| 10 | Mogilsa | 1 | Mixed woodland | 64,209738 | -21,708856 | 25^th^ August 2015 |
| 11 | Grjoteyri | 1 | Conifer plantation | 64,304316 | -21,605687 | 25^th^ August 2015 |
| 12 | Grjoteyri | 1 | Conifer plantation | 64,303423 | -21,603885 | 25^th^ August 2015 |
| 13 | Mosfellsbaer | 1 | Hiking path | 64,173118 | -21,685853 | 25^th^ August 2015 |
| 14 | Heidmork | 1 | Conifer Woodland | 64,061183 | -21,874536 | 26^th^ August 2015 |
| 15 | Vifilstadavatn | 1 | Scrub | 64,07811 | -21,87865 | 26^th^ August 2015 |
| 16 | Gudmundarlundur | 1 | Conifer plantation | 64,073916 | -21,824287 | 26^th^ August 2015 |
| 17 | Hvaleyrarvatn | 1 | Mixed woodland | 64,038497 | -21,920879 | 26^th^ August 2015 |
| 18 | Seltjorn | 1 | Conifer plantation | 63,945743 | -22,424598 | 26^th^ August 2015 |
| 19 | Selskogur | 1 | Conifer plantation | 63,871482 | -22,432698 | 26^th^ August 2015 |
| 20 | Grindavik | 1 | Sand dune coast | 63,831935 | -22,450674 | 26^th^ August 2015 |
| 21 | Hveragerdi | 2 | Public park | 64,001528 | -21,183277 | 27^th^ August 2015 |
| 22 | Hveragerdi | 2 | Conifer plantation | 64,003123 | -21,18230 | 27^th^ August 2015 |
| 23 | Olfusborgir | 2 | Cottage area | 63,995202 | -21,16466 | 27^th^ August 2015 |
| 24 | Selfoss | 2 | Mixed woodland | 63,933167 | -20,990641 | 27^th^ August 2015 |
| 25 | Selfoss | 2 | Camping site | 63,94661 | -20,992684 | 27^th^ August 2015 |
| 26 | Hella | 2 | Camping site | 63,832664 | -20,40395 | 27^th^ August 2015 |
| 27 | Fljotshlid (Tumastadir) | 2 | Mixed woodland | 63,739027 | -20,065058 | 27^th^ August 2015 |
| 28 | Fljotshlid (Tumastadir) | 2 | Mixed woodland | 63,739748 | -20,069071 | 27^th^ August 2015 |
| 29 | Fljotshlid (Tumastadir) | 2 | Mixed woodland | 63,742929 | -20,060713 | 27^th^ August 2015 |
| 30 | Skogar | 2 | Mixed woodland | 63,528378 | -19,494231 | 27^th^ August 2015 |
| 31 | Skogar | 2 | Mixed woodland | 63,529487 | -19,497149 | 27^th^ August 2015 |
| 32 | Grimsnes | 2 | Birchs woodland | 64,010961 | -20,978715 | 27^th^ August 2015 |
| 33 | Grimsnes | 2 | Birchs woodland | 64,012766 | -20,979359 | 27^th^ August 2015 |
| 34 | Ulfljotsvatn | 2 | Camping site | 64,096001 | -21,042649 | 27^th^ August 2015 |
| 35 | Klambratun | 1 | Public park | 64,136552 | -21,915654 | 28^th^ August 2015 |
| 36 | Oskjuhlid | 1 | Conifer Woodland | 64,130327 | -21,917617 | 28^th^ August 2015 |
| 37 | Bjarkarlundur | 1 | Mixed woodland plantation | 64,097107 | -21,779408 | 28^th^ August 2015 |
| 38 | Eskifjordur (Bleiksarhlid) | 3 | Camping site | 65,077776 | -14,031475 | 25^th^ Sept. 2015 |
| 39 | Neskaupsstadur | 3 | Public park | 65,148488 | -13,686997 | 25^th^ Sept. 2015 |
| 40 | Neskaupsstadur (Hjallaskogur) | 3 | Mixed woodland | 65,151569 | -13,679088 | 25^th^ Sept. 2015 |
| 41 | Faskrudsfjordur | 3 | Mixed plantation | 64,932047 | -14,021978 | 25^th^ Sept. 2015 |
| 42 | Faskrudsfjordur | 3 | Mixed plantation | 64,932016 | -14,009464 | 25^th^ Sept. 2015 |
| 43 | Egilsstadir (Tjarnargardur) | 3 | Public park | 65,264838 | -14,397078 | 26^th^ Sept. 2015 |
| 44 | Hallormsstadarskogur (Thjodskogur) | 3 | Conifer woodland | 65,133263 | -14,688127 | 26^th^ Sept. 2015 |
| 45 | Hallormsstadarskogur | 3 | Mixed woodland | 65,123379 | -14,698125 | 26^th^ Sept. 2015 |
| 46 | Hallormsstadarskogur (Kliftjorn) | 3 | Mixed woodland | 65,103167 | -14,728895 | 26^th^ Sept. 2015 |
| 47 | Hallormsstadarskogur (Hofdavik) | 3 | Camping site | 65,103167 | -14,728895 | 26^th^ Sept. 2015 |
| 48 | Hallormsstadarskogur (Jonsskogur) | 3 | Mixed woodland | 65,086574 | -14,766402 | 26^th^ Sept. 2015 |
| 49 | Hallormsstadarskogur (Guttormslundur) | 3 | Mixed woodland | 65,085838 | -14,76962 | 26^th^ Sept. 2015 |
| 50 | Hallormsstadarskogur (Ljosarkinn) | 3 | Mixed woodland | 65,08504 | -14,776043 | 26^th^ Sept. 2015 |
| 51 | Reydarfjordur | 3 | Walking path | 65,038459 | -14,212274 | 26^th^ Sept. 2015 |
| 52 | Stodvarfjordur | 3 | Mixed woodland | 64,832881 | -13,856142 | 27^th^ Sept. 2015 |
| 53 | Stodvarfjordur | 3 | Conifer plantation | 64,832318 | -13,857422 | 27^th^ Sept. 2015 |
| 54 | Djupivogur (Halsaskogur) | 3 | Conifer Woodland | 64,658544 | -14,326157 | 27^th^ Sept. 2015 |
| 55 | Hofn (Einarslundur) | 3 | Migrant passerine plantation | 64,270672 | -15,193992 | 7^th^ May 2016 |
| 56 | Hofn (Thorgeirslundur) | 3 | Decidous tree plantation | 64,266885 | -15,201836 | 7^th^ May 2016 |
| 57 | Hofn (Hrossabithagi) | 3 | Conifer plantation | 64,256429 | -15,202306 | 7^th^ May 2016 |
| 58 | Hofn (Hrossabithagi) | 3 | Conifer plantation | 64,256334 | -15,204936 | 7^th^ May 2016 |
| 59 | Stafafell (Lerkilundur) | 3 | Mixed woodland | 64,444581 | -14,936104 | 7^th^ May 2016 |
| 60 | Stafafell | 3 | Native birch woodland | 64,443288 | -14,930654 | 7^th^ May 2016 |
| 61 | Stafafell | 3 | Chirch graveyard | 64,419637 | -14,860287 | 7^th^ May 2016 |
| 62 | Hraunkot | 3 | Mixed plantation | 64,384846 | -14,802642 | 7^th^ May 2016 |
| 63 | Oskjuhlid | 1 | Conifer plantation | 64,125889 | -21,924679 | 10^th^ May 2016 |
| 64 | Fossvogsdalur | 1 | Mixed woodland | 64,117685 | -21,889117 | 10^th^ May 2016 |
| 65 | Bjarkarlundur | 1 | Mixed woodland plantation | 64,097626 | -21,780225 | 12^th^ May 2016 |
| 66 | Ellidaardalur | 1 | Mixed woodland | 64,122032 | -21,843002 | 12^th^ May 2016 |
| 67 | Mogilsa | 1 | Mixed woodland | 64,209477 | -21,708256 | 13^th^ May 2016 |
| 68 | Ulfarsfell | 1 | Conifer plantation | 64,152297 | -21,737567 | 13^th^ May 2016 |
| 69 | Fljotshlid (Tumastadir) | 2 | Mixed woodland | 63,739171 | -20,063782 | 13^th^ May 2016 |
| 70 | Fljotshlid (Tumastadir) | 2 | Mixed woodland | 63,740429 | -20,062936 | 13^th^ May 2016 |
| 71 | Fljotshlid (Tumastadir) | 2 | Mixed woodland | 63,743032 | -20,060814 | 13^th^ May 2016 |
| 72 | Oskjuhlid | 1 | Conifer Woodland | 64,131239 | -21,919129 | 29^th^ May 2016 |
| 73 | Gudmundarlundur | 1 | Conifer plantation | 64,074289 | -21,826668 | 30^th^ May 2016 |
| 74 | Hveragerdi | 2 | Public park | 64,001606 | -21,184362 | 3^rd^ June 2016 |
| 75 | Hveragerdi | 2 | Conifer plantation | 64,003268 | -21,183046 | 3^rd^ June 2016 |
| 76 | Hvolsvollur | 2 | Mixed plantation | 63,751834 | -20,213533 | 3^rd^ June 2016 |
| 77 | Selfoss | 2 | Camping site | 63,933708 | -20,991285 | 3^rd^ June 2016 |
| 78 | Selfoss | 2 | Mixed woodland | 63,945878 | -20,992758 | 3^rd^ June 2016 |
| 79 | Tjornin (Bjarkargata) | 1 | Public park | 64,143083 | -21,944876 | 21^st^ June 2016 |
| 80 | Skogar | 2 | Mixed woodland | 63,527332 | -19,492691 | 24^th^ June 2016 |
| 81 | Hveragerdi | 2 | Public park | 64,001099 | -21,183921 | 19^th^ August 2016 |
| 82 | Hveragerdi (Reykjamork) | 2 | Conifer plantation | 64,002864 | -21,181775 | 19^th^ August 2016 |
| 83 | Hveragerdi (Dynskogar) | 2 | Mixed woodland plantation | 64,003142 | -21,205141 | 19^th^ August 2016 |
| 84 | Selfoss | 2 | Mixed woodland | 63,950575 | -20,989698 | 19^th^ August 2016 |
| 85 | Selfoss | 2 | Mixed woodland | 63,945912 | -20,992765 | 19^th^ August 2016 |
| 86 | Selfoss | 2 | Camping site | 63,933698 | -20,990861 | 19^th^ August 2016 |
| 87 | Hella | 2 | Camping site | 63,831812 | -20,407024 | 19^th^ August 2016 |
| 88 | Hella | 2 | Hiking path | 63,835230 | -20,404020 | 19^th^ August 2016 |
| 89 | Hvolsvollur | 2 | Mixed woodland plantation | 63,751953 | -20,215151 | 19^th^ August 2016 |
| 90 | Fljotshlid (Tumastadir) | 2 | Mixed woodland | 63,739146 | -20,064271 | 19^th^ August 2016 |
| 91 | Skogar | 2 | Mixed woodland | 63,527625 | -19,493099 | 19^th^ August 2016 |
| 92 | Myrdalur | 2 | Farm garden | 63,444113 | -19,144321 | 19^th^ August 2016 |
| 93 | Vik (Grafargil) | 2 | Mixed vegetation,scrubs,grassland | 63,420924 | -19,008857 | 20^th^ August 2016 |
| 94 | Vik | 2 | Conifer plantation | 63,427505 | -18,906614 | 20^th^ August 2016 |
| 95 | Kirkjubaejarklaustur | 2 | Camping site | 63,792280 | -18,050620 | 20^th^ August 2016 |
| 96 | Kirkjubaejarklaustur (Skriduvellir) | 2 | Mixed woodland | 63,789801 | -18,054389 | 20^th^ August 2016 |
| 97 | Kirkjubaejarklaustur (Systrafoss) | 2 | Mixed woodland | 63,786848 | -18,059749 | 20^th^ August 2016 |
| 98 | Kirkjubaejarklaustur (Geirland) | 2 | Camping site | 63,801536 | -18,056924 | 20^th^ August 2016 |
| 99 | Mortunga II | 2 | Cottage garden | 63,840921 | -18,066085 | 20^th^ August 2016 |
| 100 | Hofn (Hrossabithagi) | 3 | Conifer plantation | 64,256464 | -15,202405 | 20^th^ August 2016 |
| 101 | Hofn (Einarslundur) | 3 | Migrant passerine plantation | 64,267461 | -15,195986 | 20^th^ August 2016 |
| 102 | Halsaskogur (Bulandsnes) | 3 | Mixed woodland | 64,658506 | -14,325978 | 20^th^ August 2016 |
| 103 | Stodvarfjordur | 3 | Mixed woodland | 64,823665 | -13,856382 | 21^st^ August 2016 |
| 104 | Faskrudsfjordur (Oseyri) | 3 | Mixed plantation | 64,936352 | -14,035789 | 21^st^ August 2016 |
| 105 | Faskrudsfjordur | 3 | Public park | 64,932003 | -14,022925 | 21^st^ August 2016 |
| 106 | Faskrudsfjordur | 3 | Mixed plantation, lupine | 64,932176 | -14,009560 | 21^st^ August 2016 |
| 107 | Eskifjordur (Bleiksarhlid) | 3 | Camping site | 65,077827 | -14,031235 | 21^st^ August 2016 |
| 108 | Neskaupsstadur (Hjallaskogur) | 3 | Mixed woodland | 65,151575 | -13,678974 | 21^st^ August 2016 |
| 109 | Reydarfjordur | 3 | Hiking path | 65,034002 | -14,205148 | 21^st^ August 2016 |
| 110 | Egilsstadir | 3 | Mixed plantation | 65,261155 | -14,398546 | 22^st^ August 2016 |
| 111 | Egilsstadir | 3 | Public park | 65,263836 | -14,397056 | 22^st^ August 2016 |
| 112 | Hallormsstadarskogur (Thjodskogur) | 3 | Conifer woodland | 65,133283 | -14,688423 | 22^st^ August 2016 |
| 113 | Hallormsstadarskogur | 3 | Mixed woodland | 65,130045 | -14,691899 | 22^st^ August 2016 |
| 114 | Hallormsstadarskogur (Hafursa) | 3 | Mixed woodland | 65,123402 | -14,698057 | 22^st^ August 2016 |
| 115 | Hallormsstadarskogur (Kliftjorn) | 3 | Mixed woodland | 65,103278 | -14,728475 | 22^st^ August 2016 |
| 116 | Hallormsstadarskogur (Hofdavik) | 3 | Camping site | 65,099944 | -14,741306 | 22^st^ August 2016 |
| 117 | Hallormsstadarskogur (Hallormsstadir) | 3 | Grazed woodland | 65,095444 | -14,738547 | 22^st^ August 2016 |
| 118 | Hallormsstadarskogur (Guttormslundur) | 3 | Mixed woodland | 65,085958 | -14,769227 | 22^st^ August 2016 |
| 119 | Egilsstadir (Selskogar) | 3 | Mixed woodland | 65,264023 | -14,379862 | 23^rd^ August 2016 |
| 120 | Seydisfjordur | 3 | Private gardens | 65,259469 | -14,010203 | 23^rd^ August 2016 |
| 121 | Myvatn (Hofdi) | 4 | Mixed woodland | 65,580017 | -16,950891 | 23^rd^ August 2016 |
| 122 | Ásbyrgi | 4 | Native birch and conifer woodland | 66,001646 | -16,513442 | 24^th^ August 2016 |
| 123 | Akurgerdi | 4 | Mixed woodland | 66,046466 | -16,439929 | 24^th^ August 2016 |
| 124 | Husavik (Husavikurfjall) | 4 | Conifer woodland | 66,048563 | -17,337862 | 26^th^ August 2016 |
| 125 | Akureyri | 4 | Grassland | 65,720461 | -18,720461 | 26^th^ August 2016 |
| 126 | Akureyri (Blomsturvellir) | 4 | Mixed plantation | 65,719932 | -18,148392 | 26^th^ August 2016 |
| 127 | Akureyri (Vaglir a Thelamork) | 4 | Conifer woodland | 65,730103 | -18,303289 | 26^th^ August 2016 |
| 128 | Akureyri (Kjarnaskogur) | 4 | Mixed woodland | 65,646837 | -18,086389 | 26^th^ August 2016 |
| 129 | Akureyri (Kristnesskogur) | 4 | Conifer woodland | 65,596076 | -18,088820 | 26^th^ August 2016 |
| 130 | Akureyri (Vaglaskogur) | 4 | Mixed woodland | 65,706978 | -17,890821 | 26^th^ August 2016 |
| 131 | Dalvik | 4 | Camping site | 65,861165 | -18,255908 | 27^th^ Agust 2016 |
| 132 | Dalvik | 4 | Farm tree plantation | 65,942301 | -18,423491 | 27^th^ Agust 2016 |
| 133 | Dalvik | 4 | Camping site | 65,963878 | -18,533956 | 27^th^ Agust 2016 |
| 134 | Varmahlid | 5 | Conifer woodland | 65,552447 | -19,445539 | 27^th^ Agust 2016 |
| 135 | Skograekt Hunvetninga | 5 | Mixed woodland | 65,659089 | -20,268258 | 27^th^ Agust 2016 |
| 136 | Hvammstangi (Bletturinn) | 5 | Mixed plantation | 65,389349 | -20,932165 | 27^th^ Agust 2016 |
| 137 | Jafnastadaskogur (Hredavatn) | 7 | Mixed woodland | 64,757772 | -21,594683 | 28^th^ August 2016 |
| 138 | Snaefellsnes (Hofstadaskogur) | 7 | Conifer plantation | 64,835288 | -22,830419 | 28^th^ August 2016 |
| 139 | Borgarnes (Skallagrimsgardur) | 7 | Public park | 64,540375 | -21,920811 | 28^th^ August 2016 |
| 140 | Skorradalur (Stalpastadir) | 7 | Conifer woodland | 64,521407 | -21,459293 | 28^th^ August 2016 |
